# Measuring Nurses’ Knowledge and Awareness of Climate Change and Climate-Associated Diseases: Systematic Review of Existing Instruments

**DOI:** 10.3390/nursrep14040209

**Published:** 2024-10-08

**Authors:** Omar Portela Dos Santos, Élodie Perruchoud, Filipa Pereira, Paulo Alves, Henk Verloo

**Affiliations:** 1 Department of Nursing Sciences, School of Health Sciences, HES-SO Valais/Wallis, University of Applied Sciences and Arts Western Switzerland, Chemin de l’Agasse 5, CH-1950 Sion, Switzerland; elodie.perruchoud@hevs.ch (É.P.); filipa.pereira@hevs.ch (F.P.); henk.verloo@hevs.ch (H.V.); 2 Faculdade de Ciências da Saúde e Enfermagem, Universidade Católica Portuguesa, Rua de Diogo Botelho 1327, 4169-005 Porto, Portugal; pjalves@ucp.pt; 3 Centre for Interdisciplinary Research in Health (CIIS—Wounds Research Lab), Universidade Católica Portuguesa, 4169-005 Porto, Portugal; 4 Service of Old Age Psychiatry, Department of Psychiatry, Lausanne University Hospital, Route de Cery 60, CH-1008 Lausanne, Switzerland

**Keywords:** eco-literacy, health literacy, nursing sciences, climate change, education, environmental health, public health, systematic review, psychometric, COSMIN, tools

## Abstract

Background: As early as 1995, the Institute of Medicine suggested that nurses were inadequately prepared for and educated about climate change and its health consequences. The aim of this systematic review is to identify the most reliable, robust, and valid instruments for measuring nurses’ knowledge and awareness of climate change and climate-associated diseases. Methods: Included studies were appraised using the Mixed-Methods Appraisal Tool and the Appraisal tool for Cross-Sectional Studies. The psychometrics and clinimetrics of the instruments were evaluated using the COSMIN Risk of Bias checklist and the COSMIN methodology for assessing content validity. Results: Medline, PubMed, Embase, CINAHL Ebesco, Cochrane Library Wiley, Web of Science Core Collection, Trip Database, JBI OVID SP, GreenFILE EBSCO, Google Scholar, ProQuest Dissertations and Theses Global, and DART-EU were consulted. The 14 studies retained identified eight different instruments evaluating attitudes, perceptions, environmental awareness, environmental sensitivity, environmental attitudes, behaviours, motivation, concern, optimism, and experience. This review is reported according to the PRISMA guidelines. Conclusions: The New Ecological Paradigm Scale (NEPS) and the Climate, Health, and Nursing Tool (CHANT) are the most reliable, robust, and valid instruments for measuring nurses’ knowledge and awareness of climate change and climate-associated diseases.

## 1. Introduction

Current data and scientific predictions about climate change suggest disaster [1,2]. A climate-change-related disaster is an event that leads to widespread damage and ecological disruption and threatens or deteriorates human life and health [3]. A disaster’s impact is determined by (i) the type of hazard itself, (ii) the vulnerability of the population affected, and (iii) the capacity to cope with its negative consequences [3]. Between 2030 and 2050, climate change is expected to cause about 250,000 additional deaths per year [4]. Since 2019, it has been recognised as a global threat to human health [5].

Human activity has led to changes in six planet-wide domains: (i) global climate change; (ii) air, water, and soil pollution; (iii) loss of biodiversity; (iv) the reconfiguration of biogeochemical cycles, particularly those of carbon, nitrogen, and phosphorus; (v) changes in land use and coverage; and (vi) the increasing scarcity of freshwater resources and arable land. These changes have negative impacts on human health and well-being and, consequently, on healthcare systems too [6].

According to the Swiss Health Observatory (Observatoire suisse de la santé, Obsan, 2016), the outpatient emergency departments (EDs) in Switzerland’s 100 largest hospitals dealt with a total of 1,722,000 cases in 2016, or 4718 admissions per day. With a minimum of 53 ED admissions per 1000 inhabitants (canton of Appenzell Innerrhoden) and a maximum of 296 admissions per 1000 inhabitants (canton of Ticino), ED cases represented 3.4% of all consultations in Switzerland [7]. In Switzerland, cantons are the country’s 26 semi-autonomous member states, each functioning with a high degree of independence. Switzerland is a federal republic, and its cantons are similar to states or provinces in other countries. They have their own constitutions, governments, laws, and tax systems. The rate of ED visits in 2011 was estimated to be 26% higher than in 2007 [8]. More than one-third (39%) of consultations involved young people aged ≤25 years, 28% involved patients aged 26–45, and 20% involved patients aged 46–56. ED services represent 9.3% of the costs of hospital outpatient services and 0.8% of total healthcare costs in Switzerland [7]. Obsan has put forward several hypotheses concerning these rates of recourse, such as a substitution with urgent recourse to office-based doctors, an inability to reconcile working hours with the opening hours of general practitioners’ offices, or the development of specific services (medical hotlines or walk-in clinics). The rising utilisation of emergency care services is a well-documented phenomenon. The health consequences of climate change are expected to exacerbate this trend, leading to a further increase in ED consultations. This poses a significant risk of overwhelming an already strained healthcare system

The three summer months of 2015, the second-hottest summer ever recorded in Switzerland, saw an estimated 2.4% increase in ED admissions, or 2768 excess cases. The highest estimates of excess ED consultations were found in the warmest regions (the canton of Ticino and the Lake Geneva region) and among people aged ≥75 years. The principal non-external causes and selected diseases were infectious and parasitic diseases (11.6%, 95%CI: 8.6–14.6%), influenza and pneumonia (11.3%, 95%CI: 6.8–15.8%), genitourinary system diseases (4.2%, 95%CI: 1.3–7.1%), and digestive system diseases (3.0%, 95%CI: 1.0–5.1%) [9]. These reasons for ED visits could be explained by heat-related disorders (e.g., dehydration, kidney failure, delirium), respiratory problems due to poor air quality, and diseases spread by flourishing microorganisms.

Raising awareness, changing behaviours, and health promotion and prevention measures are urgently needed to ensure planetary health—“the health of human civilisation and the natural systems on which it depends” [1]. The nursing discipline contributes to the population’s health and works directly to achieve the Sustainable Development Goals [10]. Nurses are often the first point of contact in healthcare systems. They are in a unique position to witness and respond to the direct health impacts of climate change and contribute to environmental health and climate-conscious care. As health professionals sensitive to patients’ vulnerabilities and emerging health needs [11], nurses are key actors in promoting planetary health by identifying climate change’s impacts on their daily practice, educating patients and communities about its health risks, and encouraging behaviours that are more environmentally sustainable. Since they advocate daily for the health and safety of individuals, families, and communities, they are strategically well positioned to respond to climate change’s impacts through their practice, research, and training. Nurses should participate in modelling and implementing health interventions to raise the population’s awareness about environmental threats, thus contributing to managing health risks.

Central concepts related to climate change and its health consequences are sustainability, eco-literacy, eco-centricity, and even eco-responsibility, all of which are vital concepts for the healthcare and nursing disciplines. Indeed, creating environmental awareness is vital for a sustainable environment and implies environmentally responsible nurses with the knowledge, skills, behaviours, and attitudes to address the climate change issues facing their profession [12] This will require nurses who can think ecologically, i.e., using eco-literate, eco-responsible, and eco-centric thinking.

The European Environment Information and Observation Network (Eionet) defines **eco-literacy**, first mentioned by Roth (1968), as the behaviours, attitudes, practices, and knowledge that society possesses concerning the maintenance, protection, or ability to take the necessary measures to maintain, restore, or improve the health of its natural resources, the ecosystem, and all external conditions affecting human health [13]. According to Roth, there are three levels of eco-literacy: (i) nominal eco-literacy, which reflects knowledge of the relevant vocabulary, without any depth; (ii) functional eco-literacy, which is the ability to use fundamental environmental knowledge, concepts, and mental skills to formulate positions for action on particular environmental issues and in everyday behaviour; and (iii) operational eco-literacy, which implies the ability to fully understand environmental issues, gather and evaluate relevant information, examine and choose between alternatives, take positions and actions to sustain and develop environmental knowledge, and use interrogative, analytical, and deductive reasoning [14].

Moreover, for nurses, **eco-responsibility** is the quality of a person, a behaviour, or an activity that considers the principle of long-term respect for the physical, social, and economic environment [15]. It involves having positive attitudes towards climate change and sustainability, a sense of responsibility, a will to change, and confidence in the future.

Finally, nurses need to make links between local actions and global consequences. They need to remove their endemic blindness, as well as their egocentric focus [16,17,18], to consider the environment beyond the immediate context of care and consider climate change from an eco-centric perspective. **Eco-centrism**, derived from the post-humanism movement, emphasises the interconnection of life forms within a complex and harmonious whole. The eco-centric perspective sees the environment and nature as an integrated community. Organisms, biological communities, and ecosystems make up the biosphere based on biospheric egalitarianism. The value system is centred on nature, as opposed to anthropocentrism, which is a human-centred value system [11].

To enable nurses to promote planetary health and adopt an appropriate stance on this issue, they must identify the level of knowledge of their ecological thinking. It is necessary and urgent, therefore, to identify the most reliable, robust, and valid instruments for measuring nurses’ knowledge and awareness of climate change and climate-associated diseases. Without assessing the current situation, we will be unable to put in place measures to contribute to the development of nurses’ eco-literacy, eco-responsibility, and eco-centricity.

Thus, this review’s research questions were as follows:

- What existing instruments are used to measure nurses’ knowledge and awareness of climate change and climate-associated diseases?
- How do the instruments identified vary in terms of their reliability, validity, and robustness for assessing nurses’ knowledge and awareness of climate change and climate-associated diseases?
- How do the instruments identified address the different aspects of nurses’ knowledge and awareness of climate change and climate-associated diseases?

## 2. Materials and Methods

The systematic review protocol was registered in the PROSPERO international database of prospectively registered systematic reviews (protocol #: CRD42023407696). It was published. It was published in International Journal of Environmental Research and Public Health [19].

This review was conducted following the recommendations of the Preferred Reporting Items for Systematic Reviews and Meta-Analyses (PRISMA) [20] COnsensus-based Standards for the selection of health Measurement INstruments (COSMIN) [21]. Systematic reviews of patient-reported outcomes (PROMs) are important for selecting the most suitable PROM to measure one’s construct of interest in a specific study population. Our research questions were directly linked to PROMs. The more nurses know about the health consequences of climate change, the more efficient, accessible, effective, and centred on patients’ needs their care will be. By better understanding climate change’s implications for health, nurses will be better prepared to assess and manage its effects on patients. Since nurses’ knowledge and awareness of climate change and climate-associated diseases affect patients’ health, they are also directly associated with PROMs. Finally, using PROMs can help nurses to gather direct information from patients on how climate change is affecting their health and well-being. These data can help develop more personalised care plans and identify patients’ specific needs related to environmental issues.

Our systematic review of PROMs involved a ten-step procedure. Steps 1 to 4 involved formulating the review’s aims, formulating eligibility criteria, performing a literature search, and selecting abstracts and full-text articles of relevant studies. Steps 5 to 8 involved evaluating the content validity and quality of eligible studies, evaluating their internal structure, evaluating the remaining measurement properties, and describing interpretability and feasibility. Finally, steps 9 and 10 involved formulating recommendations and writing and publishing the systematic review [21]. The research protocol for this systematic review has been published elsewhere [19].

### 2.1. Study Selection Criteria

#### 2.1.1. Participants and Healthcare Settings

This review considered studies involving nursing students and registered nurses (RNs) with bachelor’s, master’s, or doctoral degrees. We also included all healthcare and hospital setting categories and considered all institutions providing nursing training at the bachelor’s, master’s, and doctoral levels.

#### 2.1.2. Types of Studies

Randomised controlled trials, cluster-randomised controlled trials, non-randomised studies, observational studies, cross-sectional studies, surveys, prospective cohort studies, case–control studies, controlled before-and-after studies, interrupted time-series studies, quasi-experimental studies, and mixed-methods studies were all eligible for inclusion [22,23].

#### 2.1.3. Types of Outcome Measures

This review’s primary outcome was the identification of existing robust, validated, reliable instruments measuring nurses’ knowledge and awareness of climate change and climate-associated diseases. The review’s secondary outcomes were the measurement of nurses’ knowledge, awareness, motivation, attitudes, behaviours, beliefs, skills, and competencies regarding climate change and climate-associated diseases. We also sought out the variables that contribute to different levels of eco-literacy, as defined by the instrument.

### 2.2. Search and Process Strategies

This section enabled the implementation of steps 1–4 in the COSMIN guidelines [21].

#### 2.2.1. Search Strategy and Identification of Relevant Studies

In collaboration with a medical librarian (PM) and using predefined search terms, we conducted a systematic literature search for published articles in the following electronic databases until 30 September 2023: Medline Ovid SP (from 1946), PubMed (NOT Medline[sb]; from 1996), Embase.com (from 1947), CINAHL Ebesco (from 1937), the Cochrane Library Wiley (from 1992), Web of Science Core Collection (from 1900), the Trip Database (from 1997), JBI OVID SP (from 1998), and GreenFILE EBSCO. We also conducted a manual search of all of the relevant articles’ bibliographies and searched for unpublished studies using Google Scholar, ProQuest Dissertations and Theses Global, and DART-EUrope.eu [19].

#### 2.2.2. Study Screening and Data Extraction

**Figure 1:**
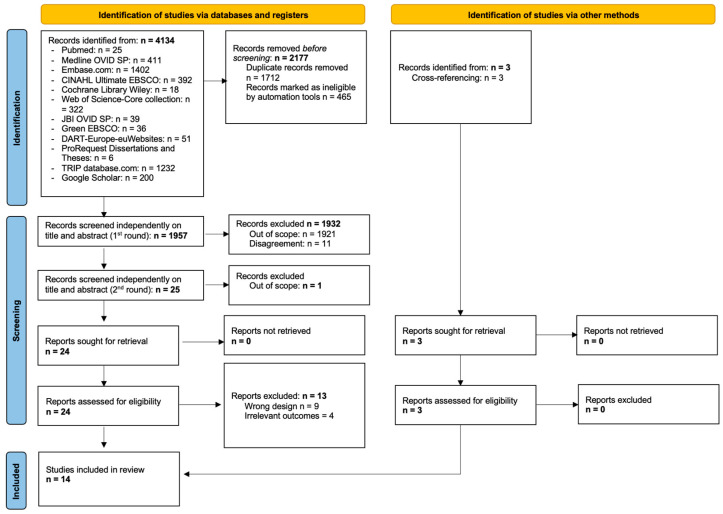
Search strategy. From Page MJ, McKenzie JE, Bossuyt PM, Boutron I, Hoffmann TC, Mulrow CD, et al. The PRISMA 2020 statement: An updated guideline for reporting systematic reviews. BMJ 2021;372:n71. doi: 10.1136/bmj.n71. For more information, visit: http://www.prisma-statement.org/ (accessed on 28 March 2024)) [20].

Two reviewers (O.P.D.S. and E.P.) independently screened the titles and abstracts identified in our searches to assess which studies met our inclusion criteria. Disagreements were resolved through discussion or, if needed, a consensus was reached after discussion with another co-author (H.V.). Duplicate records were identified using EndNote reference management software. A flowchart of the article selection process was drawn using the PRISMA statement guidelines [24] (Figure 1). Two reviewers (O.P.D.S. and E.P.) extracted the data independently using a specially designed, standardised data extraction form. Again, discrepancies were resolved through discussion and consultation with a co-author (H.V.) if necessary. The information extracted from each study included the following: (i) study authors, year of publication, and country where the study was conducted; (ii) study characteristics (including setting and design, duration of follow-up, and sample size); (iii) participants’ basic sociodemographic characteristics; (iv) characteristics of the “usual care” group; and (v) outcome measures.

### 2.3. Assessment of the Risks of Bias in the Studies Retained

Two reviewers (O.P.D.S., H.V.) independently assessed the risks of bias in all of the retained studies. Disagreements were resolved through discussion and consultation with another co-author (E.P.).

### 2.4. Statistical Analyses

Our statistical analyses followed the PRISMA guidelines [20]. Descriptive statistics were used to describe the studies and the participants involved. We computed the sum of the total population covered by the studies retained, as well as age range.

### 2.5. Methodological Quality of the Studies Retained

Two different tools were used to assess the methodological quality of the studies retained. The Mixed-Methods Appraisal Tool [22] was used to evaluate the quality of the mixed-methods studies retained [10]. We used the Appraisal tool for Cross-Sectional Studies [23] to critically appraise cross-sectional studies [25,26,27,28,29,30], descriptive studies [12,31,32,33], and descriptive survey studies [34,35,36].

### 2.6. Psychometric Quality of Tools

Psychometrics and clinimetrics are the construction and validation of measurement instruments and their assessment as valid and reliable forms of measurement [37,38]. The COSMIN taxonomy considers two sorts of measurements: (i) cross-sectional (reliability and validity) and (ii) longitudinal (reliability of change scores and responsiveness) [37].

Studies were independently assessed by two authors (O.P.D.S. and H.V.) using the COSMIN Risk of Bias checklist [39] and the COSMIN methodology for assessing the content validity (including the relevance, comprehensiveness, and comprehensibility) of PROMs [40]. The COSMIN Risk of Bias checklist is a set of questions that classify each primary study as having an “inadequate”, “doubtful”, “adequate”, or “very good” methodology for each measurement property (structural validity, internal consistency, cross-cultural validity/measurement invariance, reliability, measurement error, criterion validity, hypothesis testing for construct validity, and responsiveness) [40]. The lowest score given was taken as the total score [39,40].

## 3. Results

### 3.1. Search Strategy and Results

Our literature search retrieved a total of 4137 references. During the two rounds of our screening phase, the two researchers excluded 4123 studies. A total of 27 studies were assessed for eligibility, of which 6 were excluded. A total of 14 studies were included in this systematic review (Figure 1).

### 3.2. Characteristics of the Studies Retrieved

This review included fourteen studies: three from Europe [10,25,26]—including one from Switzerland [30]—four from the USA [26,32,34,36], six from Asia [12,27,28,29,31,33], and one from Australia [25]. Six studies used a cross-sectional design [25,26,27,28,29,30], four used a descriptive study [12,31,32,33], and one used a mixed-methods design [10]. Three studies [34,35,36] merely indicated that they were a “survey” or an “online survey”. An in-depth analysis based on the literature [41] confirmed that these studies were descriptive surveys. These types of surveys describe respondents’ attitudes, behaviours, and perceptions, and sometimes correlations between their characteristics and the system. Their designs are mainly quantitative, and the data collection, which may be cross-sectional or longitudinal, is essentially based on scales with Likert anchors [41].

The total population included in the review was composed of 7718 RNs, nursing assistant technicians, nursing administrators, faculty members, licensed practice nurses, advanced practice RNs, nursing students, and students from faculties of medicine, engineering, aquacultural engineering, economic and administrative sciences, tourism, and education. Only five studies [10,26,27,32,34] sampled only RNs and/or faculty members. The 4155 female and 1254 male participants were aged between 18 and 54 years. Three studies [25,30,35] did not report on their sample’s gender, and two studies [25,31] provided no information about their sample’s age distribution. Table 1 describes the studies retained.

### 3.3. Methodological Quality of the Studies Retained

The methodological quality of the mixed-methods study was scored as moderate (Table 2). The different domains of evaluation used in the cross-sectional descriptive studies also scored as moderate. The lowest scores were mainly awarded to methodology sections (more specifically, criteria 3 and 7 in the grid). For the results sections, criteria 13, 14, and 15 were the most frequently reported as being of medium or low quality (Table 3 and Table 4).

### 3.4. Description of the Concepts, Levels of Knowledge, Awareness, Attitudes, Motivations, Concerns, Perceptions, and Sensitivity about Climate Change, Its Health Consequences, and Sustainability

There are a few validated studies focusing on nurses’ perceptions of environmental impact, environmental awareness, and climate change. They were developed in the USA but have yet to be culturally adapted and validated in other languages [36,42].

Richardson et al. [30], Alvarez-Nieto et al. [25], and Cruz et al. [28] conducted comparative cross-sectional studies on nursing students’ attitudes regarding sustainability using the Sustainability Attitudes in Nursing Survey 2 (SANS_2). Sustainability mainly concerns the ability of healthcare professionals to provide effective care to patients despite the scarcity of resources [28]. Participants held positive attitudes toward climate change and sustainability (M = 5.472, SD = 1.05; min–max: 1–6). The highest score (M = 6.21, SD = 1.12) was for item 3, “Sustainability is an important issue for nursing”, given by students from Dalarna University (DU) in Sweden, while the lowest score (M = 4.81, SD = 1.95) was for item 4, “Sustainability should be included in the nursing curriculum”, reported by nursing students from the Catholic University of Murcia (UCAM) in Spain [25], as well as in Cruz et al.’s study (M = 6.35, SD = 1.01) [28]. However, while climate change was evaluated as an important issue for the nursing discipline (UK, M = 4.52, SD = 1.03; Germany, M = 4.93, SD = 0.94; Spain, M = 4.54, SD = 1.14; Switzerland, M = 4.65, SD = 1.12), very few students believed that issues about climate change should be included in the nursing curriculum (UK, M = 3.86, SD = 1.36; Germany, M = 4.26, SD = 1.45; Spain, M = 3.88, SD = 1.53; Switzerland, M = 4.04, SD = 1.55).

The same conclusion was applied to the concept of sustainability. The assessment of its importance to the nursing discipline scored better than the need to include it in nursing education (UK, M = 5.02, SD = 1.29; Germany, M = 5.65, SD = 1.25; Spain, M = 4.65, SD = 1.42; Switzerland, M = 5.14, SD = 1.35 versus UK, M = 4.63, SD = 1.37; Germany, M = 5.27, S = 1.34; Spain, M = 4.10, SD = 1.54; Switzerland, M = 4.61, SD = 1.45) [30]. These results are consistent with Gök et al.’s findings [31]. Indeed, the authors highlighted that the participants had moderate levels of environmental awareness (M = 3.39, SD = 0.67) and sensitivity (M = 3.54, SD = 0.69). However, a comparison between 2014 and 2019 conducted by Alvarez-Nieto et al. [25] indicated that all mean scores (SANS_2 overall and items 1 to 5) showed significantly higher values in 2019 [30,35]. In a study aiming to determine the extent to which nursing students perceived climate change’s effects on health [12], they reported that they could take effective actions to mitigate climate change’s effects on health (*p* = 0.038) and that they should be well prepared for those effects (*p* = 0.032). When asked about their sources of information about global warming and climate change, 19.6% of respondents answered “university education” [29]. Environment-related variables, such as learning about the environment and related issues in the nursing curriculum (β = −2.28, *p* < 0.001, 95%CI = −3.13, −1.43), being aware of climate change (β = −3.17, *p* < 0.001, 95%CI = −4.12, −2.22), and attending environment-related seminars and training (β = −2.23, *p* < 0.001, 95%CI = −3.08, −1.39), were all identified as significant predictors of students’ attitudes to the environment and sustainability [28,29].

In summary, knowledge about climate change and its health-related consequences was moderate among nursing students. Nevertheless, the study conducted by Sayan et al. [33] highlighted that environmental attitudes and risk perceptions among nursing students were significantly higher than among social sciences students (*p* < 0.001). Also, nursing students’ mean Environmental Attitudes Scale (EAS) score was the highest among medical, engineering, aquacultural engineering, economic and administrative sciences, tourism, and education sciences students.

**Table 1:** Characteristics of the studies retained (n = 14).

		Population			Instrument Administration			
PROM	Reference/ Design	N	Age (Mean, SD, and/or Range) in Years	Sex (n and/or %)	Country or Region	Language	Characteristics of Each PROM	Min and Max Scores/Cut-Off Point
Sustainability Attitudes in Nursing Survey (SANS_2) questionnaire	Alvarez-Nieto et al. (2022) [25] *Cross-sectional multi-site study*	846 1st-year undergraduate students from seven universities in five countries	Not reported	Not reported	UK, Spain, Germany, Sweden, and Australia	Not reported	**Outcome variable(s):** students’ attitudes towards and awareness of climate change and sustainability issues and climate change’s inclusion in nursing education **Length of the instrument:** 8 items; time for completion not reported **Response rate:** not reported	**Min–max:** not reported **Cut-off point:** not reported
New Ecological Paradigm Scale (NEPS) and Sustainability Attitudes in Nursing Survey 2 (SANS_2)	Amerson et al. (2022) [26] *Cross-sectional, descriptive study*	121 faculty teachers from 31 nursing schools	30–39 (28%) 40–49 (72%)	Male: 6 (5%) Female: 115 (95%)	South Carolina, USA	English	**Outcome variable(s):** nursing faculty’s perceptions of climate change and attitudes to adding sustainability content to the nursing curriculum. **Response rate:** 27% NEPS: **Length of the instrument:** 15 items on a 7-point Likert scale (“Strongly disagree”, “Somewhat disagree”, “Disagree”, “Neither agree nor disagree”, “Agree”, “Somewhat disagree”, and “Strongly agree”); time for completion not reported **Response rate:** not reported SANS_2: **Length of the instrument:** 5 questions on a 7-point Likert scale (“1 = Strongly disagree” and “7 = Strongly agree”); time for completion not reported	NEPS: **Min–max:** 15–105 **Cut-off point:** not reported SANS_2: **Min–max:** 5–35 **Cut-off point:** not reported
Structured questionnaire	Buriro et al. (2018) [27] *Cross-sectional study*	105 RNs from different departments at Dow University Hospital	20–29: 67 (63.8%) 30–39: 30 (28.6%) 40–49: 4 (3.8%) ≥50: 4 (3.8%)	Male: 65 (61.9%) Female: 40 (38.1%)	Pakistan	Not reported	**Outcome variable(s):** nurses’ knowledge, perceptions, and information sources about climate change **Length of the instrument:** 13 questions; time for completion not reported **Response rate:** 93.7%	Not applicable
Two tools: 1. New Ecological Paradigm Scale (NEPS) [42] 2. Sustainability Attitudes in Nursing Survey 2 (SANS_2) [29]	Cruz et al. (2018) [28] *Cross-sectional descriptive study*	280 2nd-, 3rd-, or 4th-year baccalaureate nursing students of a university in Hail City	20.03 (2.99)	Male: 123 (43.6%) Female: 157 (56.4%)	Saudi Arabia	Not reported	**Outcome variable(s):** predictors of nursing students’ attitudes towards the environment and sustainability in healthcare NEPS: **Length of the instrument:** 15 items on a 7-point Likert scale (“Strongly disagree”, “Somewhat disagree”, “Disagree”, “Neither agree nor disagree”, “Agree”, “Somewhat agree”, and “Strongly agree”); time for completion not reported SANS-2: **Length of the instrument:** 5 questions on a 7-point Likert scale; time for completion not reported **Response rate:** not reported	NEPS: **Min–max:** 15–105 **Cut-off point:** not reported SANS_2: **Min–max:** 5–35 **Cut-off point:** not reported
New Ecological Paradigm Scale (NEPS) (Part 1); Survey based on Environmental Defense Fund (EDF) work (based on Polikva et al. 2012 [34] and Streich 2014 [43]) (Part 2)	Felicilda-Reynaldo et al. (2018) [29] *Cross-sectional study*	1059 baccalaureate nursing students (registered in the 2nd, 3rd, or 4th year of bachelor’s programme)	21.39 (2.14)	Male: 443 (41.8%) Female: 616 (58.2%)	Four Arab countries (Egypt, Iraq, Palestinian Territories, and Saudi Arabia)	English	Part 1: **Outcome variable(s):** knowledge and attitudes toward climate change and its effects on health **Length of the instrument:** 15 items on a 7-point Likert scale; time for completion not reported **Response rate:** not reported Part 2: **Outcome variable(s):** knowledge, environmental attitudes, and attitudes toward health-related impacts of climate change **Length of the instrument:** 8 items, 15–25 min to complete **Response rate:** 100%	NEP scale: **Min–max:** 15–105 **Cut-off point:** not reported Survey: not applicable
Environmental Awareness and Sensitivity Scale	Gök et al. 2021 [31] *Descriptive study*	286 nursing students of a foundation university in Northern Cyprus	Not reported	Male: 103 [36] Female: 183 (64%)	Cyprus	Not reported	**Outcome variable(s):** environmental awareness and environmental sensitivity **Length of the instrument:** 37 items on a 6-point Likert scale, 15 min to complete **Response rate:** 84.1%	**Min–max:** 46–181 **Cut-off point:** not reported
Nurses’ Environmental Awareness Tool (NEAT)	Luque-Alacaraz et al. (2022) [10] *Mixed-methods study*	376 nursing staff (RNs, nursing assistant technicians, and nursing students) from Andalusia	37.7 (0.62)	Male: 101 (26.9%) Female: 275 (73.1%)	Spain	Spanish	**Outcome variable(s):** nurses’ environmental awareness **Length of the instrument:** 31 items; time for completion not reported **Response rate:** 100%	**Min–max:** 31–155 **Cut-off point:** not reported
Climate change instrument developed by Rebecca L. Franzen	May et al. (2019) [32] *Descriptive correlational study*	40 school nurses across the Commonwealth of Pennsylvania	52.76 (7.7)	Female: 40 (100%)	USA	English	**Outcome variable(s):** knowledge, attitudes, and behaviours related to the health impacts of climate change **Length of the instrument:** not reported **Response rate:** 15%	Not reported
Survey based on Environmental Defense Fund (EDF) work (based on Polikva et al. 2012 [34])	Polivka et al. (2012) [34] *Descriptive Survey*	143 public health nursing administrators and public health nurses	54 (7.8)	Male: 9 (6.3) Female: 134 (79.7)	USA	English	**Outcome variable(s):** knowledge and attitudes concerning climate change and the role of public health nurses **Length of the instrument:** 23 min **Response rate:** 22%	Not reported
Sustainability Attitudes in Nursing Survey (SANS_2)	Richardson et al. (2016) [30] *Cross-sectional design study*	916 1st-year nursing students in four different countries	Not reported	Not reported	UK, Germany, Spain, and Switzerland	German, Spanish, and French	**Outcome variable(s):** nursing students’ attitudes towards sustainability, its relevance to nursing, and its inclusion in nursing curricula **Length of the instrument:** 5 items on a 7-point Likert scale (1 = “Strongly disagree” to 7 = “Strongly agree”); time for completion not reported. **Response rate:** not reported.	**Min–max:** 5–35 **Cut-off point:** not reported
Adapted version of Richardson et al. (2016) and Sustainability Attitudes in Nursing Survey questionnaire (SANS_2)	Richardson et al. (2021) [35] *Descriptive survey*	1st-year nursing students at a university in the southwest of England 2014: n = 245 2019: n = 301	Not reported	Not reported	UK	Not reported	**Outcome variable(s):** student nurses’ attitudes towards sustainability and the climate crisis **Length of the instrument:** 8 statements on a 7-point Likert scale (1 = “Strongly disagree” to 7 = “Strongly agree”); time for completion not reported **Response rate:** not reported	**Min–max:** 5–35 **Cut-off point:** not reported
Environmental Risk Perception Scale (ERPS) and Environmental Attitudes Scale (EAS)	Sayan et al. (2016) [33] *Descriptive study*	2364 final-year students from seven faculties (Faculty of Medicine and Nursing School, Faculty of Engineering, Faculty of Aquacultural Engineering, Faculty of Economic and Administrative Sciences, Faculty of Tourism, and Faculty of Education)	21.21 (1.97) (min 18; max 32)	Male: 331 (14%) Female: 2033 (86%)	Istanbul, Turkey	Not reported	**Outcome variable(s):** nursing students’ perceptions of environmental risks and their environmental attitudes ERPS: **Length of the instrument:** 24 items on a 7-point Likert scale (1 = “It does not matter” to 7 = “It matters a lot”); time for completion not reported **Response rate:** not reported EAS: **Length of the instrument:** 21 items on a 5-point Likert scale (1 = “Strongly disagree” to 5 = “Strongly agree”); time for completion not reported. **Response rate:** not reported	ERPS: **Min–max:** 24–168 **Cut-off point:** not reported EAS: **Min–max:** 21–105 **Cut-off point:** not reported
The Climate, Health, and Nursing Tool (CHANT)	Schenk et al. (2021) [36] *Descriptive Survey*	487 -Practicing nurses: 81 -Nursing students: 255 -Faculty members: 50	35.33 (15.34)	Male: 49 (10%) Female: 438 (90%)	12 nations 93%: USA	English	**Outcome variable(s):** awareness, motivation, concern, behaviours, and optimism related to climate change and health **Length of the instrument:** items ranged from 0 to 4; number of items not reported; 10–12 min to complete **Response rate:** 100%	Not reported
Questionnaire on climate change developed by the researchers, based on the work of Korkmaz et al. (2020) [44] and Liao et al. (2019) [45]	Tuna et al. (2022) [10] *Descriptive study*	149 1st-, 2nd-, 3rd-, and 4th-year nursing students	20.58 ± 1.42 (min: 18, max: 24)	Male: 24 (162.2%) Female: 124 (83.4%)	Turkey	Not reported	**Outcome variable(s):** nursing students’ perceptions of climate change and its effects on health **Length of the instrument:** 9 questions; time for completion not reported **Response rate:** not reported	Not applicable

**Table 2:** The Mixed-Methods Appraisal Tool, version 2018.

Study	Mixed-Methods Studies (Criteria 5.1 to 5.5)	Qualitative Studies (Criteria 1.1 to 1.5)	Quantitative Descriptive Studies (Criteria 4.1 to 4.5)
Luque-Alacaraz et al. (2022) [10]	High quality Medium quality Medium quality High quality Medium quality	High quality High quality High quality Medium quality Medium quality	High quality High quality High quality Medium quality High quality

**Table 3:** Appraisal tool for Cross-sectional studies.

Study	Introduction (Criterion 1)	Methods (Criteria 2 to 11)	Results (Criteria 12 to 16)	Discussion (Criteria 17 and 18)	Others (Criteria 19 and 20)
Alvarez-Nieto et al. 2022 [25]	High quality	High quality (criteria 2, 4, 6, 7, 8, 9, 19, 11) Low quality (criterion 3) Medium quality (criterion 5)	High quality (criteria 12, 15, 16) Medium quality (criteria 13, 14)	High quality (criteria 17, 18)	High quality (criteria 19, 20)
Amerson et al. (2022) [26]	High quality	High quality (criteria 2, 4, 5, 8, 9, 10, 11) Low quality (criteria 3, 7) Medium quality (criterion 6)	Medium quality (criteria 12) Low quality (criteria 13, 14, 15)	High quality (criteria 17, 18)	High quality (criterion 19) Medium quality (criterion 20)
Buriro at al. (2018) [27]	High quality	High quality (criteria 2, 4, 5, 6, 8, 9, 10, 11) Low quality (criterion 3) Medium quality (criterion 7)	High quality (criteria 12, 13, 16) Medium quality (criteria 14, 15)	High quality (criterion 17) Low quality (criterion 18)	Low quality (criterion 19) High quality (criterion 20)
Cruz et al. (2018) [28]	High quality	High quality (criteria 2, 4, 8, 9, 10) Medium quality (criteria 3, 5, 11) Low quality (criteria 6, 7)	High quality (criteria 12, 13, 16) Low quality (criteria 14, 15)	High quality (criteria 17, 18)	High quality (criteria 19, 20)
Felicilda-Reynaldo et al. (2018) [29]	Medium quality	Medium quality (criterion 2) Low quality (criteria 3, 7, 10) High quality (criteria 4, 5, 6, 8, 9, 11)	High quality (criterion 16) Low quality (criteria 13, 14, 15)	High quality (criteria 17, 18)	High quality (criteria 19, 20)
Gök et al. 2021 [31]	High quality	High quality (criteria 2, 8, 9, 10, 11) Medium quality (criteria 3, 4, 5, 6, 7)	High quality (criteria 12, 16) Medium quality (13, 14, 15)	High quality (criteria 17, 18)	Low quality (criterion 19) High quality (criterion 20)
May et al. (2019) [32]	High quality	High quality (criteria 2, 6, 8, 9) Low quality (criteria 3, 7, 10, 11) Medium quality (criteria 4, 5)	High quality (criterion 12) Low quality (criteria 13, 14, 15, 16)	Medium quality (criterion 17) Low quality (criterion 18)	Low quality (criteria 19, 20)
Richardson et al. (2016) [30]	High quality	High quality (criteria 2, 8, 9) Low quality (criteria 3, 6, 7, 10) Medium quality (criteria 4, 5, 11)	Low quality (criteria 12, 13, 14, 15) High quality (criterion 16)	Medium quality (criterion 17) High quality (criterion 18)	Low quality (criterion 19) High quality (criterion 20)
Sayan et al. (2016) [33]	High quality	High quality (criteria 2, 5, 6, 8, 9, 10, 11) Medium quality (criterion 4) Low quality (criteria 3, 7)	High quality (criteria 12, 16) Low quality (criteria 13, 14, 15)	High quality (criteria 17, 18)	Low quality (criterion 19) High quality (criterion 20)
Tuna et al. (2022) [12]	High quality	High quality (criteria 2, 3, 4, 5, 6, 8, 9, 10, 11) Low quality (criterion 7)	High quality (criteria 12, 16) Low quality (criteria 13, 14, 15)	High quality (criteria 17, 18)	Low quality (criterion 19) High quality (criterion 20)

Criterion 1 for Introduction; Criteria 2–11 for Methods; Criteria 12–16 for Results; Criteria 17–18 for Discussion; Criteria 19–20 for Others.

**Table 4:** AXIA—Cross-sectional survey studies.

Study	Introduction (Criterion 1)	Methods (Criteria 2 to 11)	Results (Criteria 12 to 16)	Discussion (Criteria 17 and 18)	Others (Criteria 19 and 20)
Polivka et al. (2012) [34]	High quality	High quality (criteria 2, 9, 10) Low quality (criteria 3, 7, 11) Medium quality (criteria 4, 5, 6)	High quality (criteria 12, 16) Medium quality (criterion 13) Low quality (criteria 14, 15)	High quality (criteria 17, 18)	Low quality (criteria 19, 20)
Richardson et al. (2021) [35]	High quality	High quality (criteria 2, 5, 6, 8, 9) Medium quality (criteria 3, 4) Low quality (criteria 7, 10, 11)	Low quality (criteria 12, 13, 14, 16) High quality (criterion 15)	High quality (criterion 17) Low quality (criterion 18)	Low quality (criteria 19, 20)
Schenk et al. (2021) [36]	High quality	High quality (criteria 2, 8, 9) Low quality (criteria 3, 6, 7, 10, 11) Medium quality (criteria 4, 5)	High quality (criterion 12) Low quality (criteria 13, 14, 15) Medium quality (criterion 16)	High quality (criteria 17, 18)	High quality (criterion 19) Low quality (criterion 20)

Criterion 1 for Introduction; Criteria 2–11 for Methods; Criteria 12–16 for Results; Criteria 17–18 for Discussion; Criteria 19–20 for Others.

The same findings were true for RNs. Indeed, studies show that nurses have moderate knowledge, awareness, motivation, concern, self-reported behaviours at work, and self-reported behaviours at home regarding climate change and health, as well as weak or insufficient perceptions of climate change and its effects on health [27,34,36]. Even though nurses reported that they understood the impacts of climate change—such as air pollution (66.7%), poor waste management (45.7%), heat waves (37.2%), climate change (27.9%), or flooding (25.7%)—on people’s health, especially on vulnerable populations (older adults and children) [27], 55.2% of the respondents did not know what positive actions they could take [26]. These conclusions matched those in the initial survey by Schenk et al. [36] using the Climate, Health, and Nursing Tool (CHANT). The 487 nurses surveyed reported moderate levels of awareness (M = 2.97, SD = not reported, min–max: 0–4) but high levels of concern (M = 3.43, SD = not reported) about climate change’s impacts on health. Moreover, a certain number of barriers to nurses taking a position on these issues were identified, including a lack of knowledge and feelings of being overwhelmed by the topic. Indeed, they reported low levels of action on these issues in their workplaces, with a score of M = 1.81 (1 = “rarely”, 2 = “some”). Respondents’ most common sources of information about climate change’s consequences were social media (78%) and national or international media (67.6%) [26,32,34]. Moreover, the study conducted by Polivka et al. [34] found strong associations of educational levels and age with the levels of knowledge and perceptions regarding climate change’s adverse impacts on health. Indeed, new graduates and young nurses had more knowledge and stronger perceptions (*p* ≤ 0.05), and they were more likely to agree that their actions could decrease the health-related impacts of climate change (χ^2^ = 7.3, df = 2, *p* = 0.025) [34].

One study [26] used the New Ecological Paradigm Scale and the Sustainability Attitudes in Nursing Survey 2 (SANS_2) to investigate the climate change and sustainability perceptions of teachers in the nursing discipline. Over 80% of respondents did not include any content on the health implications of climate change in their classes, and over 60% did not include content on the health implications of sustainability. A significant difference was found when comparing the SANS_2 scores of teachers who included content on climate change (F(1, 119) = 20.46, *p* < 0.0001) and who included content on sustainability (F(1, 119) = 28.20, *p* < 0.0001). The SANS_2 scores (M = 26.17, SD = 5.62) of teachers who included climate change in their curricula were higher than the scores of those who did not include such content (M = 17.74, SD = 8.49). Similarly, the SANS_2 scores were higher for those who included sustainability content (M = 24.72, SD = 6.66) than for those who did not (M = 16.69, SD = 8.33). According to May et al. [32], when school nurses were asked whether they had a critical role to play in advocating for measures to address climate change, the responses fell between “agree” and “neutral” (M = 3.02, SD = 2.51, n = 40). In contrast, their attitudes about responding to climate change were favourable (M = 59.9, SD = 8.4, n = 33). This may signal ambivalence about committing to advocacy measures as teachers, although they should be leaders in promoting more knowledge of climate change’s health effects to nursing students [32].

### 3.5. Descriptions and Assessments of the Instruments

This section fulfils steps 5 to 8 of the COSMIN guidelines for systematic reviews of PROMs [21]. The 14 studies included in this systematic review used seven different instruments. The structured questionnaire used by Buriro et al. [27], the survey based on the Environmental Defense Fund (EDF)’s work employed by Felicilda-Reynaldo et al. [29] and Polivka et al.’s descriptive survey [34], and the questionnaire on climate change used by Tuna et al. [12] have not been taken into consideration, as they do not meet the criteria for using the COSMIN methodology [37,38].

Table 5 illustrates the results of the COnsensus-based Standards for the selection of health Measurement INstruments (COSMIN) checklist for the eight instruments retained.

#### 3.5.1. The Climate, Health, and Nursing Tool (CHANT)

The Climate, Health, and Nursing Tool was developed in 2019 by Schenk et al. [42]; it measures nurses’ awareness, motivation, concerns, and self-reported behaviours at work and at home. The conceptual framework that guided the CHANT’s development is the I-Change model, which emphasises that attitudes, self-efficacy, and social influences determine a person’s motivation. Thus, developing and using environmentally sustainable behaviours implies multifaceted competencies [36,42]. The descriptive study design allowed the authors to develop their items and perform an exploratory factor analysis. This exploratory factor analysis retained a five-factor model demonstrating a good model fit (comparative fit index = 0.95; root-mean-square error of approximation = 0.04; standardised root-mean-square residual = 0.09), and its items were internally consistent (Cronbach’s alpha from 0.69 to 0.89, with α = 0.70 for the awareness subscale, α = 0.89 for the concern subscale, α = 0.91 for the motivation subscale, α = 0.79 for the home behaviour subscale, and α = 0.69 for the work behaviour subscale).

The tool’s overall quality was evaluated as adequate, as measurement errors, hypothesis testing, criterion validity, and responsiveness were not reported.

In 2022, Da Woon et al. [43] established the validity and reliability of a Korean version of the CHANT, the K-CHANT. This consisted of 20 items across five domains, with two items from the original CHANT excluded. The K-CHANT’s internal consistency for all items scored a Cronbach’s alpha equal to 0.81 (α = 0.70 for the awareness subscale, α = 0.69 for the concern subscale, α = 0.69 for the motivation subscale, α = 0.58 for the home behaviour subscale, and α = 0.43 for the work behaviour subscale). The intraclass correlation coefficient between the measurement scores (0.81) indicates the instrument’s stability (awareness subscale, 0.78 (95%CI: 0.68–0.85); concern subscale, 0.66 (95%CI: 0.49–0.77); motivation subscale, 0.76 (95%CI: 0.60–0.86); home behaviour subscale, 0.78 (95%CI: 0.68–0.86); and work behaviour subscale, 0.90 (95%CI: 0.85–0.94)). Finally, the tool has been translated into Portuguese and Spanish, but we found no studies on the psychometric properties of these versions [42,43].

#### 3.5.2. Franzen’s Climate Change Instrument

A climate change instrument was developed by Rebecca Franzen (University of Wisconsin) to assess the climate change knowledge, attitudes, and behaviours of undergraduate students. It was used in a descriptive correlational study conducted by May and Noel [32].

Despite rigorous searches of the various databases, including screening over 900 articles, we found none referring to this instrument. The author was contacted on 26 October 2023 via ResearchGate, but, to date, no reply has been received. Therefore, we were unable to analyse this instrument’s quality according to the COSMIN process.

#### 3.5.3. Environmental Awareness and Sensitivity Scale (EASS)

The Environmental Awareness and Sensitivity Scale was developed by Yesilyurt et al. [45] in 2013 to assess prospective biology teachers’ environmental awareness and sensitivity. The scale comprises 37 items, subdivided into two subscales: awareness (items 1–15) and sensitivity (items 16–37). Environmental awareness is the understanding of the importance of not harming the environment, and environmental sensitivity can be defined as the willingness to take positive initiatives against environmental problems [45].

Yesilyurt et al.’s scale development study [45], conducted in Turkey, calculated the item-total correlation and performed an exploratory factor analysis to determine the construct validity. Concerning psychometric results, the scale’s initial 91 five-point Likert-type items evolved into a 37-item scale, which explained 40.683% of the variance. The Spearman–Brown formula, used to evaluate test reliability, was evaluated at 0.70. This formula helps in decision-making when considering adjustments to a test’s length, ensuring that modifications do not significantly affect the reliability of the assessment.

The overall quality of the tool was evaluated as doubtful. The tool has not been used outside the Turkish context, and its measurement error, criterion validity, and responsiveness have not been evaluated. All of the concepts covered in the study were rated at 2.

#### 3.5.4. Environmental Attitudes Scale (EAS)

The Environmental Attitudes Scale was designed by Sama in 2003 [49] to measure attitudes and beliefs related to the environment. This scale aims to quantify an individual’s perspectives and attitudes towards environmental issues [33,49]. Based on a literature review, the draft questionnaire consisted of 43 items, of which 18 were positive and 15 were negative [49]. Its pilot study was conducted on 120 students from Gazi University’s Faculty of Education. Factor analysis eliminated 21 items, with 22 items presenting factor loadings under 0.30. A reliability analysis of all of the items gave an item-total correlation above 0.24. The internal Cronbach’s alpha coefficient was 0.77.

Based on this information, the Environmental Attitudes Scale’s overall quality was evaluated as doubtful. Reliability was evaluated using internal consistency, but measurement error (rated at 2), the intraclass correlation coefficient, and Cohen’s kappa were not explored. The same applied to construct validity. Only structural validity and hypothesis testing were identified. Responsiveness was not evaluated. Finally, the tool was developed and solely applied in the Turkish context and has yet to undergo cross-cultural validation.

#### 3.5.5. Environmental Risk Perception Scale (ERPS)

Slimak and Dietz developed the Environmental Risk Perception Scale in 2006 [50]. It was adapted to Turkish by Altunoğlu and Atav in 2009 [51]. The scale was based on the value–belief–norm theory described by Stern et al. [55], which links three theoretical models—norm activation theory, the theory of personal values, and the new ecological paradigm—into a unified explanation for environmentalism. The integration of these theories leads to a hypothesised causal chain of five variables: personal values, beliefs about the ecological paradigm, awareness of consequences, ascription of responsibility, and personal norms for pro-environmental action [50]. The theory postulates that values, and especially the well-being of other humans and the biosphere (i.e., altruism), are at the core of environmental perceptions. It also suggests that general beliefs about the environment’s sensitivity to human intervention act as a filter on the plausibility of new information regarding environmental threats.

The exploratory factor analysis conducted by Maciej Serda et al. [50,51] in 2009 determined that the scale had four factors that explained 57% of the total variance. The scale’s Cronbach’s alpha internal consistency coefficient was 0.89; its reliability coefficient, calculated using the equivalent articles test method, gave a Spearman–Brown score of 0.74. To measure the perception of risk (or the awareness of consequences), the New Ecological Paradigm Scale consists of 24 items, ranging from acid rain to population growth. Factor analysis (factor loading > 0.40) revealed a four-factor solution to which we assigned the following labels: ecological scale, chemical scale, global scale, and biological scale, and they explained 61.6% of the variance [50]. The item-total correlations of all of the items were above 0.30 (from 0.3755 for item 11 to 0.6565 for item 20). The Kaiser–Meyer–Olkin value was 0.841.

The tool’s overall quality was evaluated as inadequate. Indeed, cross-cultural validity and measurement invariance were inadequate because the only information from the authors was that the 24-item risk scale developed by Slimak and Dietz in 2006 was adapted into English as the Environmental Risk Perception Scale, with some modifications [51].

#### 3.5.6. The Nurses’ Environmental Awareness Tool (NEAT)

The Nurses’ Environmental Awareness Tool is a self-reporting questionnaire designed to assess nurses’ awareness, attitudes, and behaviours concerning environmental sustainability in their professional practice. It was created and developed in the USA. It includes questions related to various aspects of environmental sustainability and healthcare, such as waste management, energy conservation, chemical management, or the environmental impact of healthcare practices. It helps identify areas where education or interventions might be necessary to promote environmentally friendly behaviours in healthcare settings [10,56].

Unfortunately, the article entitled “Psychometric Properties of the Nurses’ Environmental Awareness Tool”, published in 2016 [10,52], was not accessible in any of the databases consulted, nor was it possible to order it from our media library. A message was sent directly to the author via the ResearchGate interface on 23 October 2023, but, to date, no response has been received. Therefore, it was impossible to evaluate this tool according to the COSMIN process.

In 2022, Luque-Alcaraz et al. [10] validated a Spanish version of the questionnaire, named NEAT-es. Its consistency and validity indicated similar results to the original.

#### 3.5.7. New Ecological Paradigm Scale (NEPS)

Dunlap and Van Liere developed the NEPS in 1978 [57] to measure adherence to the new ecological paradigm—a concept from environmental sociology and psychology representing a worldview or belief system regarding human interaction with the environment. The NEPS assesses a person’s beliefs and attitudes toward the environment, addressing concepts such as anthropocentrism versus eco-centrism, which examine human dominance over nature and the limits to growth [50,53]. It consists of a series of items to which respondents indicate their level of agreement or disagreement. High scores indicate a more eco-centric, environmentally conscious worldview, while low scores suggest a more anthropocentric, human-centric perspective regarding the environment.

The initial tool consisted of 12 items and three distinct dimensions (balance of nature, limits to growth, and human domination of nature), and it had good internal consistency (Cronbach’s alpha = 0.81). In 2000, it was modified to move away from the notion of “human exceptionalism”, which suggested that humans were exempt from the constraints of nature. Moreover, the emergence of ozone depletion, climate change, and human-induced global environmental change had highlighted the importance of including items focusing on potentially catastrophic environmental changes. The new 15-item scale (including 6 from the original NEPS and 4 very slightly modified) was constructed to achieve these purposes [53]. The new version showed internally consistent measurement instruments. Indeed, the correlations were strong, ranging from 0.33 to 0.62. It also presented more internal consistency than the original version (Cronbach’s alpha of 0.83 versus 0.81). Moreover, the new version explains 31.3% of the total variance between the items. The multitrait–multimethod approach suggested that the new NEPS possessed predictive validity (r = 0.61). Finally, the new version has also been used to examine the environmental orientations of ethnic minorities in the USA and residents of other nations such as Canada, the Baltic states, Turkey, and Japan.

The NEPS is the most widely used social psychological measure in the literature on environmentalism. Its overall quality was evaluated as adequate. Only its reliability and measurement error have not been clarified. Concepts such as content validity, structural validity, and criterion validity were rated as “very good”.

#### 3.5.8. Sustainability Attitudes in Nursing Survey (SANS_2)

The initial Sustainability Attitudes in Nursing Survey (SANS) was piloted and psychometrically evaluated across different European countries, such as the UK, Spain, and Germany. It was developed at Plymouth University in the UK in 2016 to evaluate nursing students’ attitudes to sustainability. The tool is based on social constructivism, and the development of its items was based on group discussions between experts in nursing education and sustainability [30]. The SANS questionnaire was revised so that SANS_2 focused on five Likert-scale items. Correlational analysis of the English-language version revealed that all five items showed positive and highly significant (*p* < 0.001) Pearson correlations. In addition, the item-total correlations were all high and positive (Item 1, 0.75; Item 2, 0.83; Item 3, 0.83; Item 4, 0.84; Item 5, 0.52). Finally, the five items loaded on a single factor that explained 58% of the variance [28,33]. The questionnaire was revised (based on the pilot-phase results) and translated into German, Spanish, Dutch, French, Swedish, and Arabic [54]. However, searches of the various databases found no published articles.

The SANS_2 is an appropriate instrument with which to assess sustainability-related attitudes among nursing students, even though the tool’s structural validity and overall quality were rated as doubtful.

## 4. Discussion

This systematic review aimed to identify the most reliable, robust, and valid instruments for measuring nurses’ knowledge and awareness of climate change and climate-associated diseases. To achieve this objective, we followed the Preferred Reporting Items for Systematic Reviews and Meta-Analyses (PRISMA) and the ten-step COSMIN procedure for performing systematic reviews of PROMs. This work identified eight different tools: the Climate, Health, and Nursing Tool (CHANT), Franzen’s climate change instrument, the Environmental Awareness and Sensitivity Scale (EASS), the Environmental Attitudes Scale (EAS), the Environmental Risk Perception Scale (ERPS), the Nurses’ Environmental Awareness Tool (NEAT), the New Ecological Paradigm Scale (NEPS), and the Sustainability Attitudes in Nursing Survey 2 (SANS_2). Based on the COSMIN checklist, all of these tools showed different results in terms of internal consistency, reliability, measurement error, content validity, structural validity, hypothesis testing, cross-cultural validity, criterion validity, and responsiveness.

The eight tools retained use different terms to illustrate the concept of “knowledge and awareness”, including attitudes, perceptions, environmental awareness, environmental sensitivity, environmental attitudes, behaviours, motivations, concerns, optimism, and experience. The terms used to illustrate the concept of “climate change and climate-associated diseases” were sustainability, climate crisis, and environmental risk issues.

Steps 9 and 10 of the systematic reviews of PROMs involve the formulation of recommendations and, finally, reporting on the systematic review [21]. The two instruments with the best ratings for their study’s methodological quality of measuring PROMs were the CHANT and NEPS tools, as shown in Table 5.

The CHANT was the first comprehensive survey to assess nurses’ awareness, experience, motivations, and self-reported behaviours related to climate change and health [42]. The overall rating quality of its measurement properties indicated that the instrument was adequate. It also showed good psychometric properties [42,43,44] and was adapted into Korean [43]. Regarding the COSMIN Risk of Bias checklist, the elements that were not explored were measurement error (reliability), hypothesis testing and criterion validity (construct validity), and responsiveness (Table 5)

The concepts making up the subdomains of the CHANT are awareness, concern, motivation, and action, and they are emphasised as responses to climate change. Adapting and responding to climate change requires government policies and voluntary efforts at an individual level. Also, at a time of increasing awareness about climate change, nurses need to recognise its impacts on health and act as advocates for their patients. To prepare them for roles as leaders in this domain of health, nursing education should integrate climate change issues into its curricula. Nurses are the first line in health education. They play a key role in addressing the health impacts of climate change by direct patient care, client and community education, education, advocacy, and health policy development. As recommended by Neal-Boylan et al. [58] and McDermott-Levy et al. [59], integrating climate change topics into nursing curricula is vital for preparing a workforce capable of addressing the health challenges posed by climate change. The CHANT highlights the gap between practice and education [42,43,44].

The CHANT has numerous advantages. It assesses awareness, factors that motivate people to action (or demotivate them), and behaviours related to climate change action. This provides essential information for developing future education, practice, and programmatic efforts aimed at the nursing profession. Also, one of the CHANT’s principal strengths is that, despite being developed for nurses, it can be used to measure the cognitive behavioural levels of other healthcare professionals. This has become an essential feature since The Lancet Countdown emphasised that positively responding to climate change would only be possible through multidisciplinary collaboration [60]. A better understanding of the relationships between awareness, motivations, and behaviours may help inform leaders in the field of nursing about how to support nurses more effectively concerning climate advocacy. The CHANT could also serve as an educational tool, providing nurses with information and resources to understand climate change’s impacts on health. It facilitates actions by encouraging nurses to adopt proactive stances to address climate-related health issues; it empowers them to advocate for policies and practices that promote environmental sustainability, public health, and resilience to the impacts of climate change. The CHANT could promote resilience in healthcare systems and communities by giving them knowledge about this problem. Indeed, the CHANT provides guidelines and resources for incorporating climate and health topics into patient education, healthcare policies, and professional nursing standards. Thus, nurses can become advocates for environmental health, engaging and working collaboratively with communities on climate and health issues to promote sustainable practices and mitigate health risks associated with climate change [36]. Although the tool has many advantages, there are a few limitations that should be considered. Effectively, the answers may be biased. Since it is a self-administered instrument, nurses may overestimate their knowledge or report more favourable attitudes and behaviours due to social desirability bias. This could lead to skewed results, which may not accurately reflect their actual competencies or practices in relation to climate change and health. Also, the tool may not be designed to track changes in knowledge, attitudes, or behaviours over time, since no repeated measurements over time have been carried out to date. This limits its ability to assess whether educational interventions or training programs improve nurses’ climate health literacy in the long term. Finally, the CHANT might not fully capture the cultural variations in how climate change affects health or how nursing practices adapt in the cultural context of Switzerland. For use in Switzerland, and to reflect different socio-cultural determinants of health and climate vulnerabilities, it has to be translated and culturally adapted.

The New Ecological Paradigm Scale (NEPS) is a widely used tool in environmental sociology and psychology. It was designed to measure individuals’ attitudes and beliefs regarding the environment. It categorises individuals based on their environmental worldview, ranging from more anthropocentric (human-centred) to more eco-centric (nature-centred) perspectives [45]. The overall rating of the measure’s properties indicated that the instrument was of adequate quality. Of the eight tools evaluated in this systematic review, the NEPS was the tool that covered the most elements from the COSMIN Risk of Bias checklist. Indeed, the only two elements that it does not explore are reliability and measurement error (Table 5).

The items on the NEPS cover several key themes related to environmental attitudes, such as the relationship between humans and nature (assessing whether individuals view humans as separate from nature or as part of the ecological whole), sustainable resource use (exploring beliefs about resource consumption, whether individuals believe resources are limited, and the need for sustainable practices), and environmental protection (evaluating attitudes toward environmental conservation efforts, such as the importance of protecting ecosystems and biodiversity).

The NEPS has numerous advantages. It provides a structured way of gauging an individual’s concerns about the environment. Researchers can understand the depth and nature of an individual’s environmental attitudes by analysing their responses to its items. It can also identify trends and patterns in environmental attitudes within specific demographics, such as age groups, genders, or cultural backgrounds. This information can guide targeted environmental education or policy initiatives. Studies have shown that attitudes measured using the NEPS can be indicative of subsequent environmentally responsible behaviour. This predictive quality can help in designing interventions or campaigns aimed at fostering pro-environmental actions. The NEPS’s results can inform policymakers about the prevailing environmental attitudes within a population, helping to develop policies that are aligned with public sentiment and needs. Finally, this scale has been adapted and used in a variety of cultural contexts, enabling comparative studies on environmental attitudes and perceptions across different societies (Canada, Sweden, the Baltic states, Japan, Spain, and Latin America) [45]. Despite all of these advantages, the tool also has a few points to consider. First of all, it is mainly used in the context of sociology and psychology, and less in the nursing field. Moreover, the studies did not report cut-off points or the total length of completion. This can lead to some ambiguity in interpretation. Indeed, certain statements about the balance of nature or the limits to growth may be interpreted differently based on the respondent’s prior knowledge or ideological stance. Additionally, the NEPS measures general environmental attitudes rather than specific behaviours or actions. It may not capture how individuals translate their environmental beliefs into practical, everyday actions, which may limit its ability to predict environmentally responsible behaviour. Finally, like the CHANT, the NEPS has not been yet adapted to the Swiss cultural context. To use it with Swiss nurses, it will have to be translated and culturally adapted.

Eco-literacy, eco-responsibility, and eco-centricity are crucial competencies to reduce the direct and indirect negative effects of climate change. Even though climate change poses a serious threat to human health, healthcare organisations and nurses have been relatively slow to engage with it. Different studies [61,62] have identified several social, cultural, and psychological barriers that may have led to inconsistencies between environmental issues and nursing practice, as well as a lack of engagement. Six main themes were identified: sustainability, endemic blindness to global issues, environmental numbness, social norms, assigning the priority to sustainability, and a psychology of responsibility and blame. To better understand psychological barriers, it is necessary to evaluate nurses’ knowledge and awareness of climate change and climate-associated diseases using the most reliable, robust, and valid instruments. A reliable, validated tool to measure nurses’ awareness and behaviours related to climate change is needed, and the CHANT and the NEPS might fill this gap.

One final aspect worth noting is that the participants in eight [12,25,28,29,31,33,35,54] of the 14 studies selected were students, perhaps because they are the future of the nursing profession and will be living and working with the effects of climate change for a long time. It is time to integrate environmental literacy into nursing curricula. To assess its relevance and efficiency, we need to establish students’ levels of knowledge on the subject. The earlier in their curriculum students attend lessons on this topic, the better. Indeed, in Felicilda-Reynaldo’s study [28], second-year nursing students reported more positive environmental attitudes than fourth-year students. Nursing students were more aware of climate change and its impact on public health than students in other faculties. It is this generation of future carers that the public is counting on to deal with this problem.

### 4.1. Systematic Review’s Strengths

To the best of our knowledge, no previous systematic literature reviews have been conducted to identify the most reliable, robust, and valid instruments for measuring nurses’ knowledge and awareness of climate change and climate-associated diseases. Another strength of this review is that it included six cross-sectional design studies, seven descriptive design studies, and one mixed-methods design study. The fourteen studies retained allowed researchers to analyse the psychometric properties of eight different tools using the COSMIN Risk of Bias checklist. Furthermore, we attentively followed highly recommended methodological norms and guidelines, making our findings an extremely reliable overview of the different tools available, with precise analyses of their strengths and weaknesses.

### 4.2. Systematic Review’s Limitations

This systematic review may nevertheless present some limitations. First, despite a thorough literature search using recognised methodological guidelines and recommendations, and although we used many different terms to describe our main concepts of interest, we may have missed some studies that met all of our selection criteria due to study search errors or investigators’ mistakes. Second, despite this review’s worldwide scope, some language and publication bias may still be present. Finally, another limiting aspect of this systematic review is that two out of the eight instruments measuring nurses’ knowledge and awareness of climate change and climate-associated diseases (the NEAT and the climate change instrument by Rebecca L. Franzen) could not be evaluated.

### 4.3. Implications for Practice and Research

If we wish to ensure that the most reliable, robust, and valid instruments for measuring nurses’ knowledge and awareness of climate change and climate-associated diseases truly add value to their profession, researchers will have to assess their cultural and contextual relevance in the diverse settings in which nursing education and practice occur. Secondly, to ensure that the nursing profession is aware of these issues and adopts an appropriate professional stance, raising awareness and implementing appropriate changes will be necessary. The changes discussed in this review include adding climate change and sustainability issues to nursing curricula. However, implementation of these innovations must consider the specific characteristics and needs of the caregivers involved, the uniqueness of their clinical setting, and their medical speciality. Thus, educational settings and future workplaces must be assessed with the chosen and adapted tool in mind. The starting point should be nurses’ knowledge and beliefs about climate change and its impacts on people’s health. Only then should proposals for change be put in place, in collaboration with all of the stakeholders. Implications for practice and research must consider these processes to ensure success.

## 5. Conclusions

This systematic review closely evaluated eight different scales and finally recommends the use of the two with the best values calculated using the COnsensus-based Standards for the selection of health Measurement INstruments (COSMIN). Today’s practising nurses are inadequately prepared for and educated about climate change and its consequences on health. The first step to addressing this issue is investing in undergraduate and continuing education to include more information about environmental health and climate-conscious care. Nurses need to have a comprehensive knowledge of environmental health issues to perform their individual and professional duties to the best of their ability. Indeed, nurses’ challenge is not only to be more aware and knowledgeable about the climate’s impact on health but also to maintain and adapt their expertise by implementing changes in practice. To see whether nurses have the necessary expertise to offer, their eco-literacy will have to be assessed using an appropriate, reliable, robust, and validated tool. Environmental education is the key to becoming aware of climate change and being an agent of change in attitudes towards it.

## Figures and Tables

**Table 5:** Results of the COnsensus-based Standards for the selection of health Measurement INstruments (COSMIN) Checklist.

			Reliability			Validity					Responsiveness		Recommendations
							*Construct Validity*						
Tool	Study Reference	Description (Subdimensions/Scales)	Internal Consistency	Reliability	Measurement Error	Content Validity	Structural Validity	Hypothesis Testing	Cross-Cultural Validity	Criterion Validity	Responsiveness	Comments	
The Climate, Health, and Nursing Tool (CHANT)	[36,42,43,44]	1. Awareness 2. Concern 3. Motivation 4. Home behaviour 5. Work behaviour	3	3	---	3	3	---	4	---	---	- Good model fit (comparative fit index = 0.95, root-mean-square error of approximation = 0.06, standardised root-mean-square residual = 0.04) - Cronbach’s alphas 0.69 to 0.89 - Cross-cultural: Korea	A
Climate change instrument developed by Rebecca L. Franzen		Three subscales: 1. Attitudes are assessed using a 5-point semantic differential scale 2. Knowledge is based on the number of correct responses 3. Behaviour is scored using a 5-point Likert scale	---	---	---	---	---	---	---	---	---	---	---
Environmental Awareness and Sensitivity Scale (EASS)	[31,45,46,47,48]	Two subscales: 1. Environmental awareness subscale including ozone layer, air pollution, waste disposal, and recycling 2. Environmental sensitivity scale, including developments in the environment, participation in environmental activities and events, and reactions to people who pollute - Min–max scores: 46–181	2	2	---	2	2	2	---	---	---	- Cronbach’s alpha for the EASS = 0.921 - Cronbach’s alpha for the awareness subscale = 0.912 - Cronbach’s alpha for the sensitivity subscale = 0.902 - Total score correlation from 0.000 to 0.674 - Kaiser–Meyer–Olkin = 0.786 > 0.70 - Spearman–Brown = 0.70 - Cross-sectional: Cyprus	B
Environmental Attitudes Scale (EAS)	[33,49]	- 10 items ranked positively, and 11 items ranked negatively - Min–max scores: 21–105	3	---	---	---	3	2	---	---	---	- Cronbach’s alpha = 0.77 - Factor loadings ≥ 0.30 - Item-total correlation > 0.24	B
Environmental Risk Perception Scale (ERPS)	[33,50,51]	Four subscales: 1. Ecological risks 2. Chemical waste risks 3. Global environmental risks 4. Risks of resource depletion or loss of biodiversity	3	3	2	---	3	3	1	---	---	- Factor loadings ≥ 0.40 - Cronbach’s alpha for ERPS = 0.94 - Cronbach’s alpha for the ecological risks subscale = 0.91 - Cronbach’s alpha for the chemical waste risks subscale = 0.89 - Cronbach’s alpha for the risks of resource depletion subscale = 0.64 - Cronbach’s alpha for global environmental risks = 0.88 - Spearman–Brown = 0.74 - Item-total correlations of all items ≥ 0.30 (from 0.3755 for item 11 to 0.6565 for item 20). - Kaiser–Meyer–Olkin = 0.841.	C
Nurses’ Environmental Awareness Tool (NEAT)	[10,52]	Three subscales: 1. NAS: Nurse Awareness Scales (11 items) 2. NPEB: Nurse Professional Ecological Behaviours Scales (9 items) 3. PEB: Personal Ecological Behaviours Scales (11 items)	---	---	---	---	---	---	3	---	---	- Cross-cultural validity: Spain	---
New Ecological Paradigm Scale (NEPS)	[26,53]	Five aspects of the environment: 1. Reality of limits to growth 2. Anti-anthropocentrism 3. Fragility of nature’s balance 4. Rejection of the idea that humans are exempt from the constraints of nature 5. Possibility of an eco-crisis or ecological catastrophe	3	---	---	4	4	3	3	4	3	- Cronbach’s alpha = 0.83 - Item-total correlations from 0.33 to 0.62 - All items load from 0.40 to 0.73 - Multitrait–multimethod, r = 0.61 - Cross-cultural validity: Canada, Sweden, Baltic states, Japan, Spain, Latin America	A
Sustainability Attitudes in Nursing Survey (SANS_2) questionnaire	[25,26,30,54]	1. Climate change for nursing 2. Incorporation of climate change issues in nursing curricula 3. Sustainability for nursing 4. Incorporation of sustainability issues in nursing curricula 5. Sustainability in personal life 6. Learning about the impact of climate change as a nursing student 7. Concerns about the environment: choice of university 8. Influence of the university’s sustainability reputation	3	---	---	---	2	3	3	---	---	- English version’s Cronbach’s alpha = 0.82 - German version’s Cronbach’s alpha = 0.73 - Item-total correlations (Item 1, 0.75; Item 2, 0.83; Item 3, 0.83; Item 4, 0.84; Item 5, 0.52) - Item intercorrelations ranged from 0.28 to 0.80 - Cross-cultural validity: German, Spanish, and French, Sweden, and five Arabic-speaking counties	B

Key: 4 =very good; 3 = adequate; 2 = doubtful; 1 = inadequate, not reported; ---: not rated; A = recommended; B = promising; C = insufficient.

## Public Involvement Statement

There was no public involvement in any aspect of this research.

## Guidelines and Standards Statement

The Preferred Reporting Items for Systematic Reviews and Meta-Analyses (PRISMA) and the ten-step COSMIN procedure for performing systematic reviews of PROMs.
